# Cell-Based Assay Development and Standardization for Antibody–Drug Conjugates

**DOI:** 10.3390/biotech15040096

**Published:** 2026-10-20

**Authors:** Dengyun Sun, Amy Hsu, Hu Long, Ke Qin, Jonathan Welch, Scott McCann, Zhijie Jey Cheng

**Affiliations:** 1 Large Molecule Analytical, Merck & Co., Inc., 126 East Lincoln Avenue, Rahway, NJ 07065, USA; 2 Sichuan Kelun-Biotech Biopharmaceutical Co., Ltd., 666 Xinhua Avenue, Wenjiang District, Chengdu 611138, China; 3 Department of Process Research and Development, Merck & Co., Inc., 126 East Lincoln Avenue, Rahway, NJ 07065, USA

**Keywords:** antibody–drug conjugate, cell-based assay, specificity, ready-to-use

## Abstract

ADC-111 is an Antigen-1-targeting antibody–drug conjugate with approximately eight payloads conjugated to one monoclonal antibody. The primary mechanism of action of ADC-111 is payload-induced cytotoxicity after antigen–antibody binding-mediated internalization. A cell-based assay using T24 cells engineered with Antigen-1 expression was developed and phase-appropriately validated to measure the potency of ADC-111 drug substance and drug product. This specific and stability-indicating assay detected potency loss resulting from degradation in the complementarity-determining region of the antibody, and correlated closely with the results from the Antigen-1 binding enzyme-linked immunosorbent assay, demonstrating that the ADC-111 cell-based assay is antigen-binding dependent. The potency measured by the cell-based assay also showed good correlation with varying drug–antibody ratio values, but ELISA did not. Notably, the ready-to-use format of the cell-based assay had performance comparable to that of the continuous culture using the same procedure, allowing both formats to be used interchangeably. In addition, the same parental cell line T24 was engineered with other tumor antigens, and the corresponding cell-based assay also showed good specificity, demonstrating the proof-of-concept of using a single parental cell line for multiple ADCs to standardize and accelerate cell-based assay development.

## 1. Introduction

Antibody–drug conjugates (ADCs) are a class of precision medicines made of a monoclonal antibody (mAb) covalently conjugated to a cytotoxic payload via a chemical linker [1,2,3,4,5,6,7,8]. The mAb portion of the ADC recognizes the target antigen on the tumor cells, facilitating its entry into the cells through endocytosis. Upon internalization, the cytotoxic payload is released into the cytoplasm, resulting in apoptosis of cancer cells [9]. The above canonical mechanism of action (MoA) serves as an overall framework for ADCs. There are other potential MoAs such as bystander effector, antibody-dependent cellular cytotoxicity (ADCC), antibody-dependent cellular phagocytosis (ADCP) and complement-dependent cytotoxicity (CDC), which differ appreciably for different ADCs [10,11,12]. While ADCs were designed to limit the toxicity caused by chemotherapeutic agents through improved tumor targeting, severe adverse events have been observed in many ADC clinical trials [13]. The efficacy and toxicity of an ADC depend on the antibody, the linker, the payload, and the complex interactions with the tumor microenvironment [8,9,10,11,12,13]. Progress in synthetic biochemistry, including mAb production, linker technology and novel payloads, has paved the way for a new generation of ADCs with the potential to improve the activity and toxicity profiles, therefore ushering a new wave of ADC approvals [14,15,16,17,18,19]. Currently, there are at least 15 ADCs approved globally and hundreds of clinical trials ongoing [20,21,22,23,24]. Many ADCs have shown impressive activities, therefore occupy an increasingly important position in tumor therapy.

While demonstrating impressive efficacy in cancer treatment, ADCs present significant challenges for chemistry, manufacturing, and controls (CMC) due to the complexity of the molecules [25,26,27]. Composed of both small-molecule and large-molecule components, ADCs require additional points of analytical control and intensified characterization [25,26,27]. The potency of an ADC, typically measured by a cell-based assay (CBA), is one of the critical quality attributes (CQAs) in the release and stability specifications of both drug substance (DS) and drug product (DP). Unlike antigen-binding assays such as enzyme-linked immunosorbent assay (ELISA) and surface plasmon resonance (SPR), a CBA measures the biological consequences of the ADC binding to the target cells. Therefore, a CBA is expected by regulatory agencies as a key analytical control for ADC clinical development. On the other hand, developing a specific and robust CBA that reflects the MoA of the ADC and reliably detects subtle structural and functional changes in the ADC remains highly challenging and requires significant lead time.

ADC-111 is an Antigen-1 targeting antibody-drug conjugate with approximately eight copies of topoisomerase I inhibitor and cleavable linker conjugated to one monoclonal antibody via the inter-chain disulfide bonds. Antigen-1 plays a critical role in cell adhesion, proliferation, migration, and immune-modulatory signaling. Overexpression of Antigen-1 has been found in various malignancies. As a result, Antigen-1 has emerged as an attractive target for systemic therapy in cancer. In this study, we described the development and phase-appropriate validation of an ADC-111 CBA using a T24-Antigen-1+ cell line. The ADC-111 CBA is highly sensitive to both antigen binding and variations in drug-to-antibody ratio (DAR). We also evaluated the ready-to-use (RTU) assay format, which demonstrated performance comparable to that of continuous culture. Furthermore, we demonstrated the proof-of-concept of using one parental cell to engineer different target antigens to standardize and accelerate CBA development for ADCs.

## 2. Materials and Methods

### 2.1. Cell Line Engineering

The T24-Antigen1+ stable cell line was generated using lentiviral transduction. T24 cell line (HTB-4) was purchased from ATCC (Manassas, VA, USA). A plasmid containing Antigen-1 coding sequence was purchased from Sino Biological (Beijing, China). The full length gene of Antigen-1 was cloned into a lentivirus vector and the packaged lentivirus was used to transduce T24 cells. The monoclonal cell line with stable expression of Antigen-1 was selected by limited dilution. The surface expression of Antigen-1 on the T24-Antigen-1+ cell line was verified with an approximately 4 × 10^4^ Mean fluorescence intensity by flow cytometry (Beckman, Shanghai, China). A two-tier cell banking system consisting of a master cell bank (MCB) and working cell bank (WCB) was established. Both MCB and WCB were frozen with cryopreservation media CryoStar^®^ CS10 (StemCell Technologies, Cambridge, MA, USA) and stored in liquid nitrogen. T24-Antigen1+ cells were maintained in McCoy’s 5A Medium (Thermo Fisher Scientific, Waltham, MA, USA) containing 10% fetal bovine serum (FBS) (Cytiva, Marlborough, MA, USA), 100 U/mL of Penicillin-Streptomycin (Thermo Fisher Scientific, Waltham, MA, USA), 25 mM HEPES (Thermo Fisher Scientific, Waltham, MA, USA), and 3 µg/mL puromycin (InvivoGen, San Diego, CA, USA). The assay medium contains the above components except for puromycin. T24-Antigen-2 and T24-Antigen-3 stable cell lines were generated and maintained similarly.

### 2.2. ADC-111 Materials, Forced Degradation and Varying DAR Samples

ADC-111 reference standard (RS) used for CBA development and the ADC-111 used to prepare the forced degradation samples were manufactured by Sichuan Kelun-Biotech Biopharmaceutical Co., Ltd., Chengdu, Sichuan, China. To generate the forced degradation samples, ADC-111 was stressed by AAPH (2,2′-Azobis(2-amidinopropane) dihydrochloride), heat, or light stress conditions. AAPH stress was performed by mixing a 70-fold molar ratio of AAPH to ADC-111. Heat stress was conducted at 40 °C over 24 h. Light stress samples were staged at 10 °C according to International Council for Harmonization (ICH) guidelines Q1B for 0.5x, 1.0x, and 2.0x light stress conditions [28]. ADC-111 with varying DAR samples were generated by adjusting the ratios of the reducing agent (TCEP (tris(2-carboxyethyl)phosphine) and the linker–payload relative to the antibody during the reduction and conjugation process to achieve the target DAR values. After the conjugation was complete, samples were quenched by addition of 4 M acetic acid. The crude quenched conjugation reaction was purified by removing excess TCEP, payload, and linker–payload by passing 1 mL of quenched conjugation solution through a Zeba^TM^ spin desalting column, 7K MWCO (Thermo Fisher Scientific, Waltham, MA, USA) that had previously been conditioned with 20 mM histidine buffer (pH 6.0).

### 2.3. ADC-111 CBA

T24-Antigen1+ cells were seeded in white 96-well plates at a density of 3 × 10^3^ cells per well in 150 µL of assay medium and allowed to adhere for 1–4 h. ADC-111 RS and test samples were prepared in duplicate with an initial concentration of 1000 ng/mL followed by 2-fold serial dilutions down to 7.813 ng/mL in assay medium. Fifty microliters of the above-prepared dilutions were added to the seeded cells at a 1:4 volume ratio, resulting in a final concentration range of 250 ng/mL to 1.953 ng/mL. After 70–74 h of incubation, CellTiter-Glo reagent (Promega, Madison, WI, USA) was added to the 96-well plates, and the luminescence was measured using a microplate reader (Molecular Devices, San Jose, CA, USA). Relative potency (%RP) was determined relative to the ADC-111 RS by applying parallel line analysis to the dose–response curves using SoftMax Pro 7.0 (Molecular Devices, San Jose, CA, USA). For the RTU format, one vial of T24-Antigen1+ WCB was thawed, counted, and used directly in the assay following the same procedure as above. ADC-222 and ADC-333 CBA using correspondingly engineered T24 cells were performed in a similar manner.

### 2.4. ADC-111 CBA Phase Appropriate Validation

To assess the ADC-111 CBA method performance, six samples corresponding to the target potency levels of 200%, 141%, 100%, 71%, 50%, and 35% of the reference standard were tested by three analysts on two separate days (6 runs in total) with two potency data generated per sample per run (N = 12 at each target level). ADC-111 CBA was appropriately validated following the guidelines from ICH Q2 (R2) [29] and USP <1033>, Biological Assays Validation [30]. The assessment of the relative accuracy, linearity and precision was performed using the individual potency values from the six runs in SAS JMP 16, similar to the previous description [31,32]. Relative accuracy was expressed as the percent relative bias of each target potency level. The %relative bias and its 90% confidence interval (CI) were calculated using the following equations: %relative bias = $100\times \left(\frac{\mathrm{Estimated}\;\mathrm{Potency}}{\mathrm{Target}\;\mathrm{Potency}}-1\right)$; 90% lower %relative bias = $100\times \left(\frac{\mathrm{Lower}\;90\%\;\mathrm{CI}\;\mathrm{of}\;\mathrm{Estimated}\;\mathrm{Potency}}{\mathrm{Target}\;\mathrm{Potency}}-1\right)$; 90% upper %relative bias = $100\times \left(\frac{\mathrm{Upper}\;90\%\;\mathrm{CI}\;\mathrm{of}\;\mathrm{Estimated}\;\mathrm{Potency}}{\mathrm{Target}\;\mathrm{Potency}}-1\right)$. A linearity regression was performed by plotting the estimated potency as the dependent variable against target potency as the independent variable on a logarithmic scale. Linearity was assessed by the %proportional bias according to the equation % proportional bias = 100 × (2*^b^*^−1^ − 1), where b is the estimated slope from the linear regression. Precision measures the overall variability under a variety of normal testing conditions within one laboratory. It includes repeatability (within-run variability) and intermediate precision (sum of within-run and between-run variability). A variance component analysis was performed on the natural log-transformed potency for each potency level and all levels combined to estimate between-run and within-run variance. The variance estimates for repeatability and intermediate precision were converted back to the original scale and expressed in terms of relative standard deviation (%RSD). The repeatability %RSD was calculated using the following equation: Repeatability %RSD = $100\times \sqrt{e^{{\hat{\sigma }}_{\mathrm{intra}}^{2}}-1};$ and the intermediate precision %RSD was calculated using the following equation: Intermediate Precision %RSD = $100\times \sqrt{e^{{\hat{\sigma }}_{\mathrm{intra}}^{2}+{\hat{\sigma }}_{\mathrm{inter}}^{2}}-1}$, where ${\hat{\sigma }}_{\mathrm{intra}}^{2}$ refers to within-run variance, and ${\hat{\sigma }}_{\mathrm{inter}}^{2}$ refers to between-run variance.

### 2.5. ADC-111 ELISA

ADC-111 ELISA was used to measure the antigen-binding activity of ADC-111. Briefly, recombinant human Antigen-1 protein (Acro Biosystems, Beijing, China) was coated onto 96-well microplates. Serially diluted ADC-111 RS and test samples were added to the coated plates and incubated to allow binding. Following incubation, goat anti-human IgG Fcγ-HRP antibody (Jackson ImmunoResearch Laboratories, West Grove, PA, USA) was added for detection, followed by addition of LumiGLO peroxidase chemiluminescent substrate (SeraCare, Milford, MA, USA). The luminescence signal was measured using a microplate reader (Molecular Devices, San Jose, CA, USA). The %RP of ADC-111 by ELISA was calculated and reported similarly to ADC-111 CBA as described above.

### 2.6. DAR Measurement

The DAR of ADC-111 was determined using reversed-phase liquid chromatography. Samples were treated with TCEP to reduce the ADC into heavy- and light-chain components. H-Class Bio UPLC (Waters, Milford, MA, USA) equipped with a MabPac reversed-phase column (Thermo Fisher Scientific, Waltham, MA, USA) and a Photodiode Array (PDA) detector (Waters, Milford, MA, USA) was used for chromatographic analysis. Reversed-phase gradient with increasing percentage of acetonitrile was used to elute components of the ADC based on their differences in hydrophobicity, with more hydrophobic components having longer retention times. Due to the hydrophobic nature of the payload, longer retention times correspond to higher drug loading on both the heavy- and light-chain components. The average DAR was calculated as the weighted average of the integrated peak areas of individual species.

## 3. Results

### 3.1. ADC-111 CBA Specificity

To evaluate the specificity of ADC-111 CBA, an isotype ADC with the same linker–payload and target DAR, ADC-111 mAb, free payload and unconjugated linker–payload were compared with ADC-111 in the CBA. The antibody in the isotype ADC molecule is directed to a viral protein with no endogenous expression in mammalian cells; therefore, it serves as a good isotype control. For better comparion, the linker–payload and payload concentrations used in this experiment were 7.8x that of ADC-111, which is equimolar considering the DAR of 7.8. As shown in Figure 1A, the mAb portion of ADC-111 did not exert cytotoxicity, while the free payload had slightly lower cytotoxicity than ADC-111, confirming that the cytotoxicity of ADC-111 arises from the payload component, not the mAb component. The unconjugated linker–payload had weaker cytotoxicity compared with free payload and ADC-111. The isotype ADC with the same linker–payload and target DAR but no human target showed minimal cytotoxicity, demonstrating that the cytotoxicity of ADC-111 is antigen-binding dependent. To further confirm the specificity of ADC-111 CBA, serially diluted ADC-111 was added to T24-Antigen-1+ cells and Antigen-1-negative parental T24 cells seeded on the same assay plates following the ADC-111 CBA procedure. As shown in Figure 1B, ADC-111 exerted minimal cytotoxicity in parental T24 cells, confirming that ADC-111 CBA is specific to Antigen-1.

### 3.2. CBA Stability Indicating Property and Correlation with ELISA

To demonstrate that ADC-111 CBA is stability-indicating, ADC-111 forced-degraded samples were tested by CBA and compared with ADC-111 ELISA. As shown in Table 1, while there was no potency loss by heat stress, AAPH and 0.5x light stress caused significant potency loss in both ELISA and CBA, consistent with the increased oxidation levels at HC-W37 and LC-W94 within the complementarity-determining region (CDR). Light stress of 1.0x or above caused severe degradation, with no valid data for DAR, ELISA or CBA generated. Under AAPH stress, there was only a minor drop in DAR from 7.8 to 6.7 (87% of control), which is not expected to be the major contribution to the potency loss in the CBA. The potencies by ELISA and CBA were compared in Table 1 and the %Control (normalized by T0) was plotted in Figure 2. The relative potencies determined by CBA and ELISA showed close agreement (coefficient of determination R^2^ = 0.96; slope = 0.99). The similar level of potency loss by CBA and ELISA confirms that CBA is antigen-binding dependent and covers ELISA.

### 3.3. CBA and DAR Correlation

To further investigate the structure-function relationship and evaluate the biological impact of conjugation, ADC-111 samples with target DAR of 2, 4, 6, and 7 in duplicate and an additional sample matching the RS target DAR of 7.8 (same as ADC-111 reference standard) were generated from the conjugation process. Residual free payload levels in all preparations were confirmed to be below the qualification limits. Analysis of a representative subset of samples by ELISA confirmed that antigen binding remained unaffected by the conjugation process. The samples with varying DAR were evaluated for actual DAR and cell-based cytotoxicity. As shown in Figure 3, the %RP determined by CBA demonstrated good linear correlation with the experimentally determined DAR (R^2^ = 0.99). Data from the varying DAR study demonstrated that the ADC-111 CBA is reflective of DAR variations.

### 3.4. ADC-111 CBA Phase Appropriate Validation

The ADC-111 CBA was phase-appropriately validated in a good manufacturing practice (GMP) laboratory. The validation results, including specificity, linearity, relative accuracy, precision, and range, are summarized in Table 2. Figure 4 shows the linearity (Figure 4A) and relative accuracy (Figure 4B) results. ADC-111 CBA had good linearity with a coefficient of determination R^2^ = 0.98 and slope = 0.95 in the testing range of 35% to 200% target potency. The proportional bias was estimated as −3.5% (Table 2). The %relative bias estimates at each target level with their corresponding 90% confidence intervals (CI) were plotted in Figure 4B. The largest %relative bias was 8.7% at the 35% target potency with a 90% CI (4.6, 13.0). The overall repeatability %RSD was 6.9%, and the intermediate precision %RSD was 7.5%, based on all samples combined.

### 3.5. ADC-111 CBA in RTU Format

To simplify ADC-111 CBA workflow, the ready-to-use (RTU) format of T24-Antigen-1+ cells was evaluated. In the traditional continuous culture (CC) approach, a vial of WCB is thawed and cultured a couple of days prior to setting up the CBA. For RTU format, the same WCB is thawed, counted, and used directly in the CBA without prior expansion. As shown in Figure 5A,B, both the CC and RTU formats yielded nearly identical dose–response curves, assay windows, and EC_50_. More importantly, the assay performance was highly comparable between RTU and CC as well (Table 3). These promising results demonstrated the feasibility of integrating both RTU and CC formats in a single method, offering operational flexibility for testing laboratories.

### 3.6. CBA Using T24 Cell Engineered with Other Tumor Antigens

Following the success of the T24-Antigen-1+ cell line in ADC-111 CBA, the same parental cell line, T24, was engineered with other tumor antigens, Antigen-2 and Antigen-3, to support ADC-222 and ADC-333 CBA, respectively. Both ADC-222 and ADC-333 had similar payload and the same target DAR as ADC-111, while ADC-444 was included as an isotype control. The experimental procedures for ADC-222 and ADC-333 CBA were similar to ADC-111 CBA, with optimization for the incubation time. As demonstrated in Figure 6A,B, both CBAs using T24-Antigen-2+ and T24-Antigen-3+ cells yielded good dose–response curves and assay specificity. These results successfully validate the proof-of-concept of using a single parental cell line for multiple ADC CBAs, establishing a standardized approach that can significantly accelerate CBA development timelines.

## 4. Discussion

In this study, a CBA using T24-Antigen-1+ cells for ADC-111 was developed and phase-appropriately validated with good specificity, linearity, relative accuracy, precision, and range. In general, specificity is challenging for ADC CBA for several reasons. First, membrane-permeable payloads and linker–payloads also exert cytotoxicity. It is even more challenging for ADCs with unstable linkers, such as ADC-111. Second, the measurement of cell viability, which is typically used for ADC CBA, is not specific to ADCs. To improve the specificity of ADC CBA, the target antigen expression needs to be high enough to differentiate from non-antigen-dependent killing. Despite such challenges, ADC-111 CBA using T24 cells engineered with Antigen-1 expression demonstrated great antigen-dependent cytotoxicity. Another key consideration for ADC CBA is the choice of the cell line. Oftentimes, screening of tumor cells for target antigen expression is needed, which is highly resource-intensive. Even after a tumor cell line with high antigen expression is identified, sometimes native tumor cell lines could exhibit inconsistent growth rates, or the slow growth of the tumor cell line could lead to long incubation times for the assay. In this study, we demonstrated a streamlined approach for ADC CBA by engineering one parental cell line with different antigens for different ADCs with similar payloads. This approach can help standardize and accelerate ADC CBA development. Note that the choice of the parental cell line may vary for different categories of payload.

Based on a recent Biophorum survey, RTU cells are used in potency assays by 80% of the respondent companies at some stage of assay development [33]. Utilization of RTU cells in bioassays can save time and resources, reduce the risk of contamination from maintaining continuous cell culture, and increase the scheduling flexibility for sample testing [33,34,35]. In addition, an RTU bank represents a uniform cell population and passage number that can mitigate the risk of cellular changes that could occur in continuous cell culture over time, therefore simplifying laboratory-to-laboratory assay transfer. However, it has been challenging to implement RTU for biological potency assays in general. First, not all cell lines are suitable for RTU format, possibly related to the growth characteristics, cell modifications or receptor expression recovery issues. Second, in some cases, a larger cell number, longer incubation time, or both were needed for RTU than for the continuous culture format, resulting in different method procedures between RTU and continuous culture. Interestingly, ADC CBA is particularly suitable and easy to implement in the RTU format, likely due to the low number of cells needed and long incubation time allowing cell recovery. The logistics of implementing RTU, including the size of the RTU bank, cryopreservation media, cell density, cost, storage space, and stability, need to be considered and may vary for different cell lines.

Whether an antigen-binding assay is needed or not for an ADC release and stability panel is still a highly debated topic within the ADC industry. In a recent publication [26], the authors recommended that a binding assay may be included in the early clinical stage when a CBA is not yet available, but a CBA should be included for DS and DP control for late clinical phase and commercialization. It is well known that the primary MoA of ADC is cytotoxicity through antigen binding, internalization, and release of payload, which kills the tumor cells. A binding assay such as ELISA only captures the first step of the events, while CBA detects the endpoint, cell death; therefore, it is more MoA-reflective. For ADC-111, the stressed conditions mainly impacted the CDR, and the potency loss was reflected by both ELISA and CBA. In the case of DAR variants without impacting the CDR, potency loss was reflected by CBA but not ELISA. ADC-111 CBA has demonstrated stability-indicating capability across both the mAb component and conjugation-related CQAs, rendering the binding ELISA redundant. Based on comprehensive data collected from release, stability, and forced degradation studies, exclusion of binding ELISA from the ADC-111 release and stability panel can be scientifically justified.

## Figures and Tables

**Figure 1:**
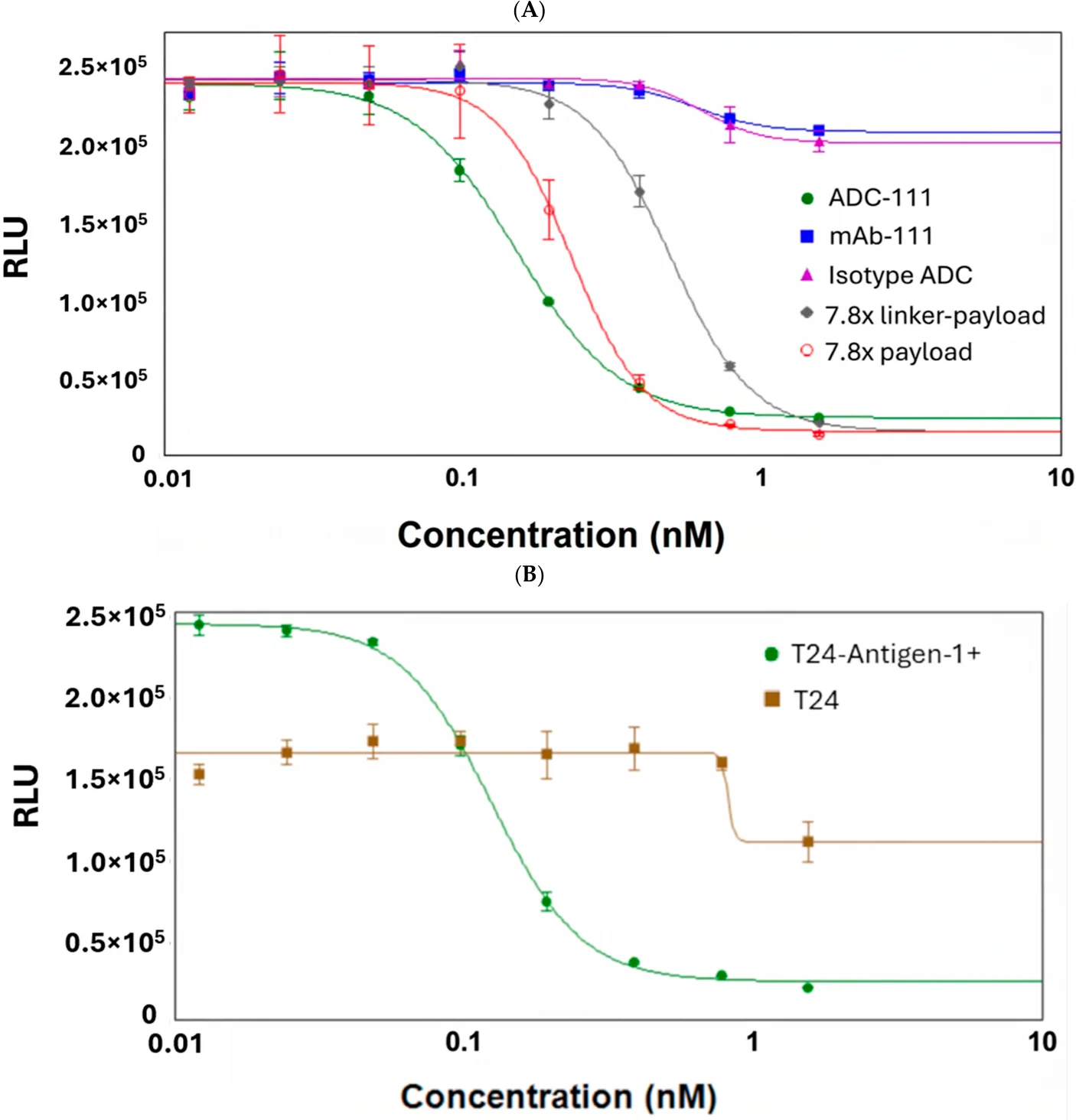
ADC-111 CBA Specificity. (**A**) The equimolar concentrations (nM) of 7.8x free payload and 7.8x drug-linker, mAb-111, and an isotype ADC with similar DAR were compared with ADC-111 in the CBA. (**B**) ADC-111 dose–response curves were compared between T24-Antigen-1+ cells and T24 parental cells. RLU: relative luminescence unit. Error bars represent the standard deviation of *N* = 2 measurements.

**Figure 2:**
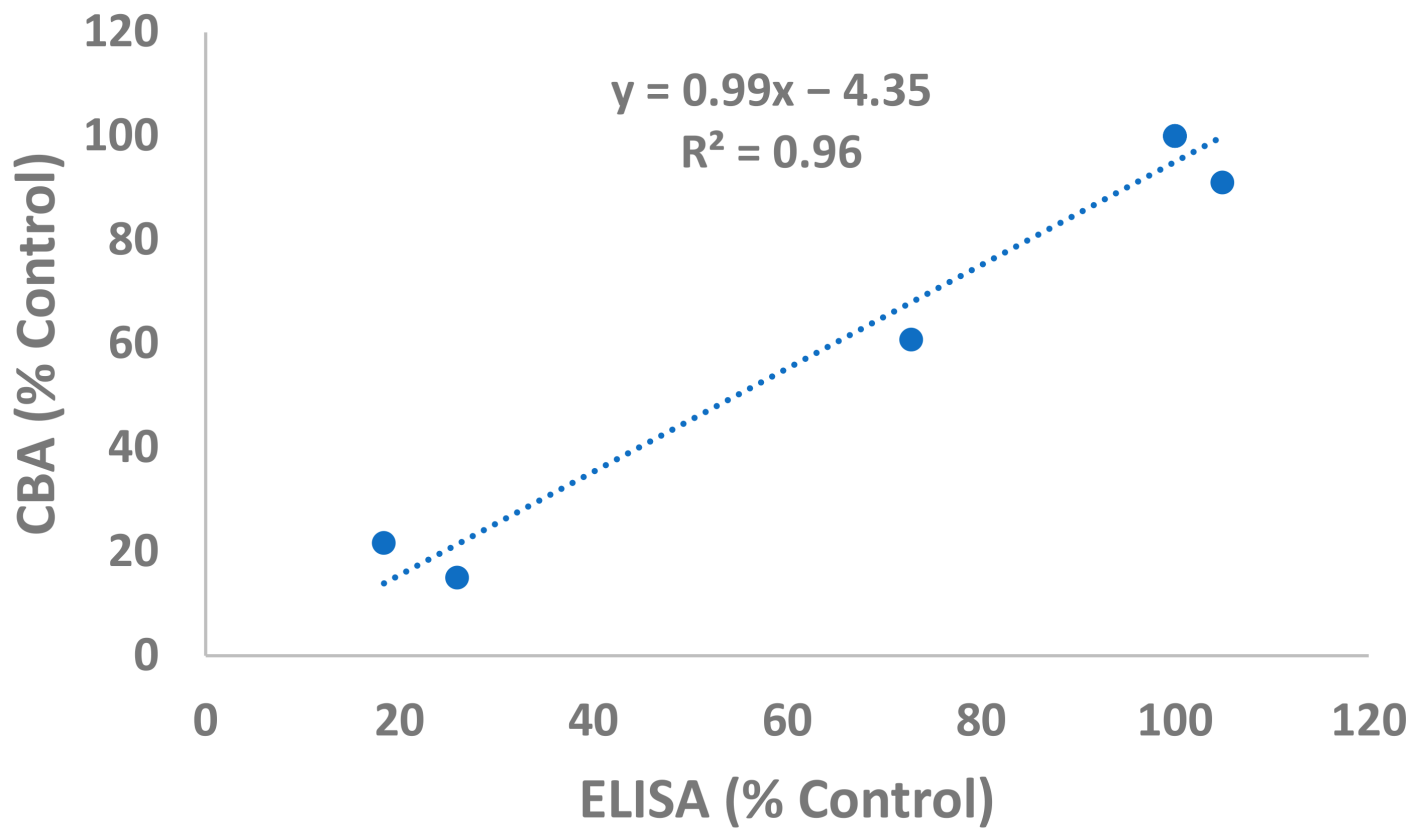
CBA and ELISA correlation analysis. The CBA (%Control) was plotted against the ELISA (%Control). Seven samples (N = 1 reportable potency per sample) were used for regression analysis using Microsoft Excel. The blue, dashed line was the fitted line. The linear regression equation and R^2^ were displayed on the graph.

**Figure 3:**
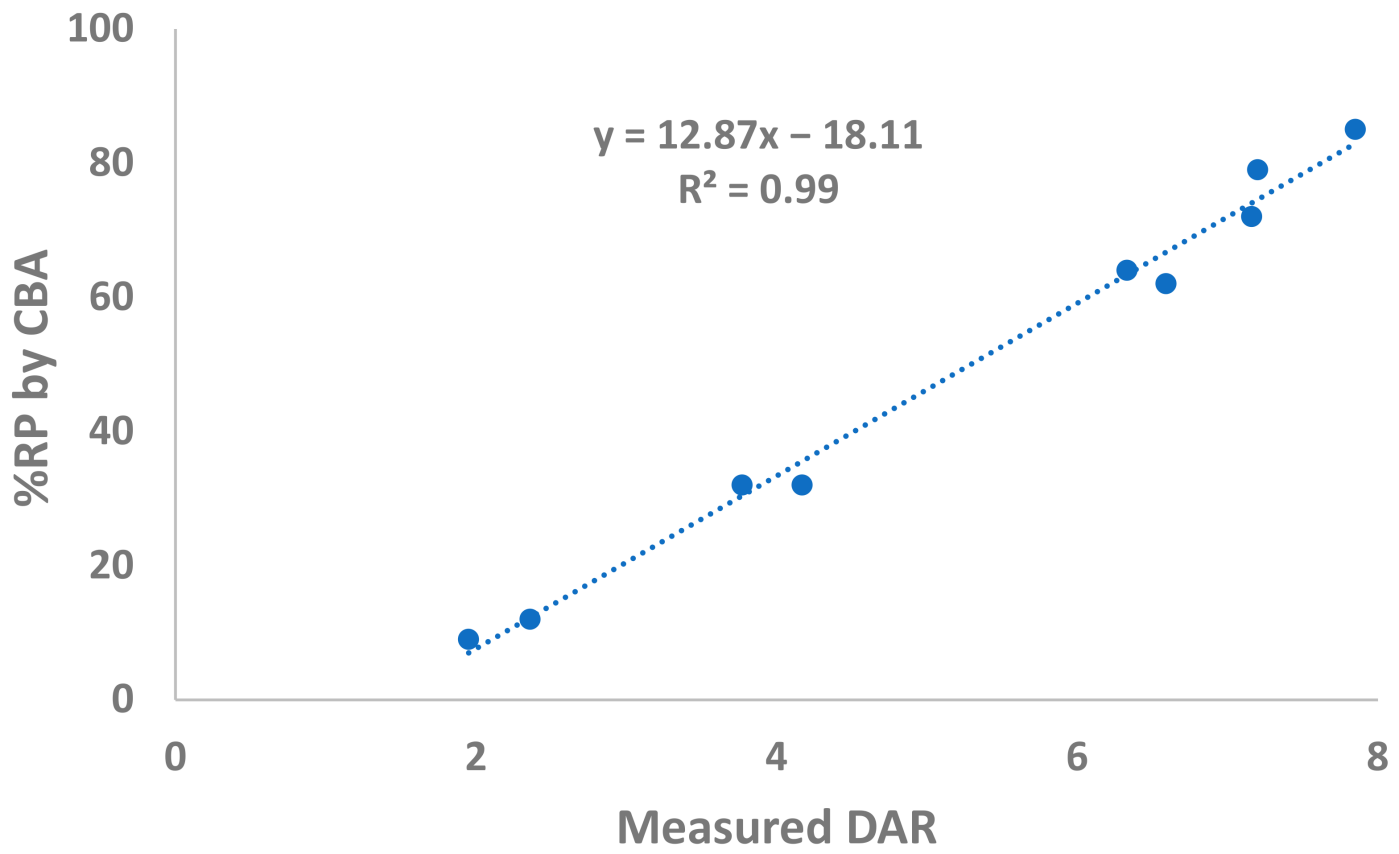
CBA and DAR correlation analysis. The %RP by CBA was fitted against the measured DAR values. Nine samples (N = 1 reportable potency per sample) were used for regression analysis using Microsoft Excel. The blue, dashed line is the fitted line. The linear regression equation and R^2^ were displayed on the graph.

**Figure 4:**
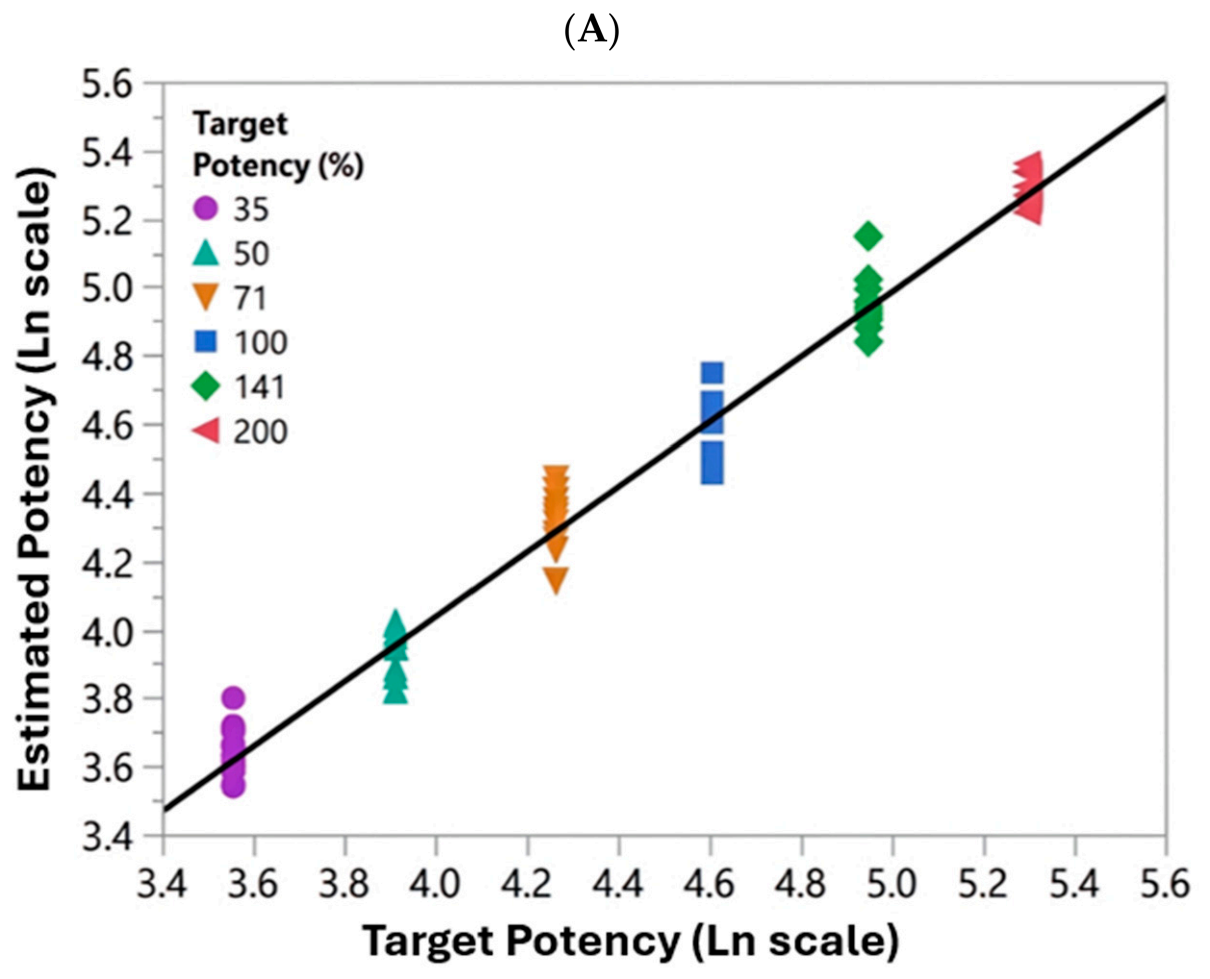

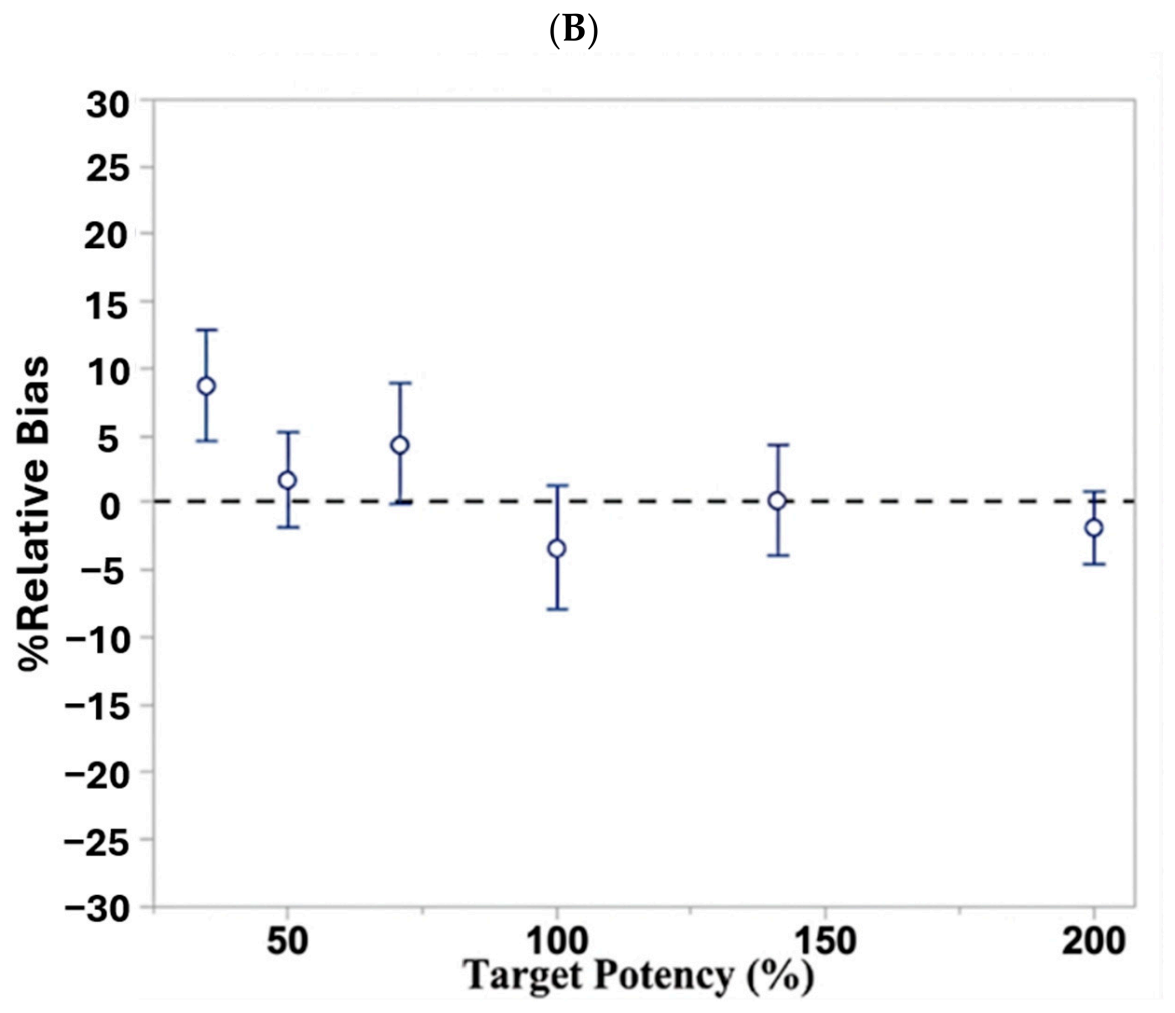
Linearity and relative accuracy of ADC-111 CBA. (**A**) Linearity of ADC-111 CBA in the range of 35–200% target potency on a logarithmic scale. *X*-axis: target potency on a logarithmic scale. *Y*-axis: estimated potency on a logarithmic scale. Black line: fitted line. (**B**) %Relative bias of ADC-111 CBA. Circle: %relative bias estimates. Error bar: 90% CI. Dashed line: y = 0.

**Figure 5:**
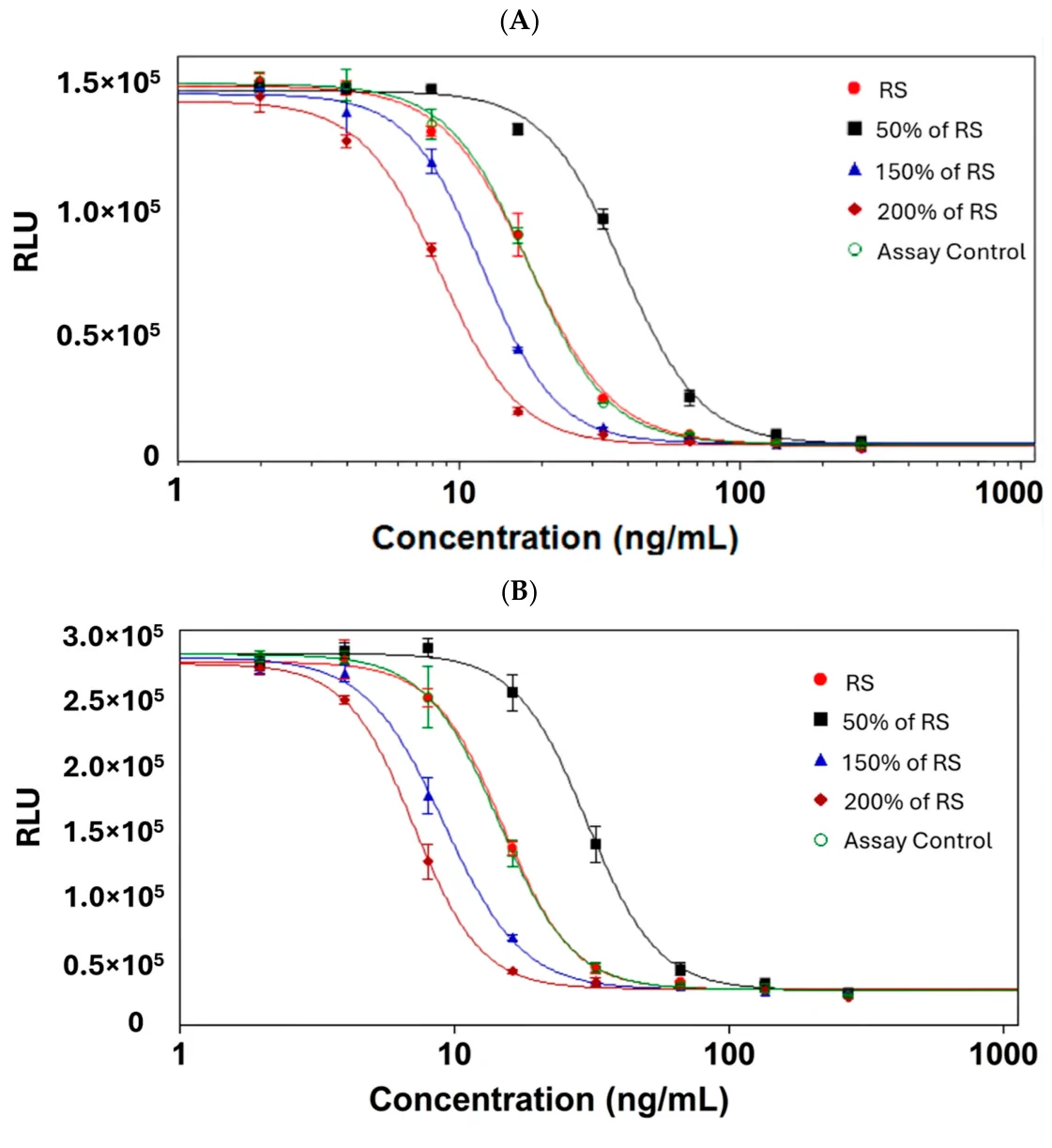
ADC-111 CBA using RTU. (**A**) Representative curves of ADC-111 CBA using CC format. (**B**) Representative curves of ADC-111 CBA using RTU format. Assay Control is the same as RS with independent dilutions. RS: reference standard; RLU: relative luminescence unit; CC: continuous culture; RTU: ready-to-use. Error bars represent the standard deviation of N = 2 measurements.

**Figure 6:**
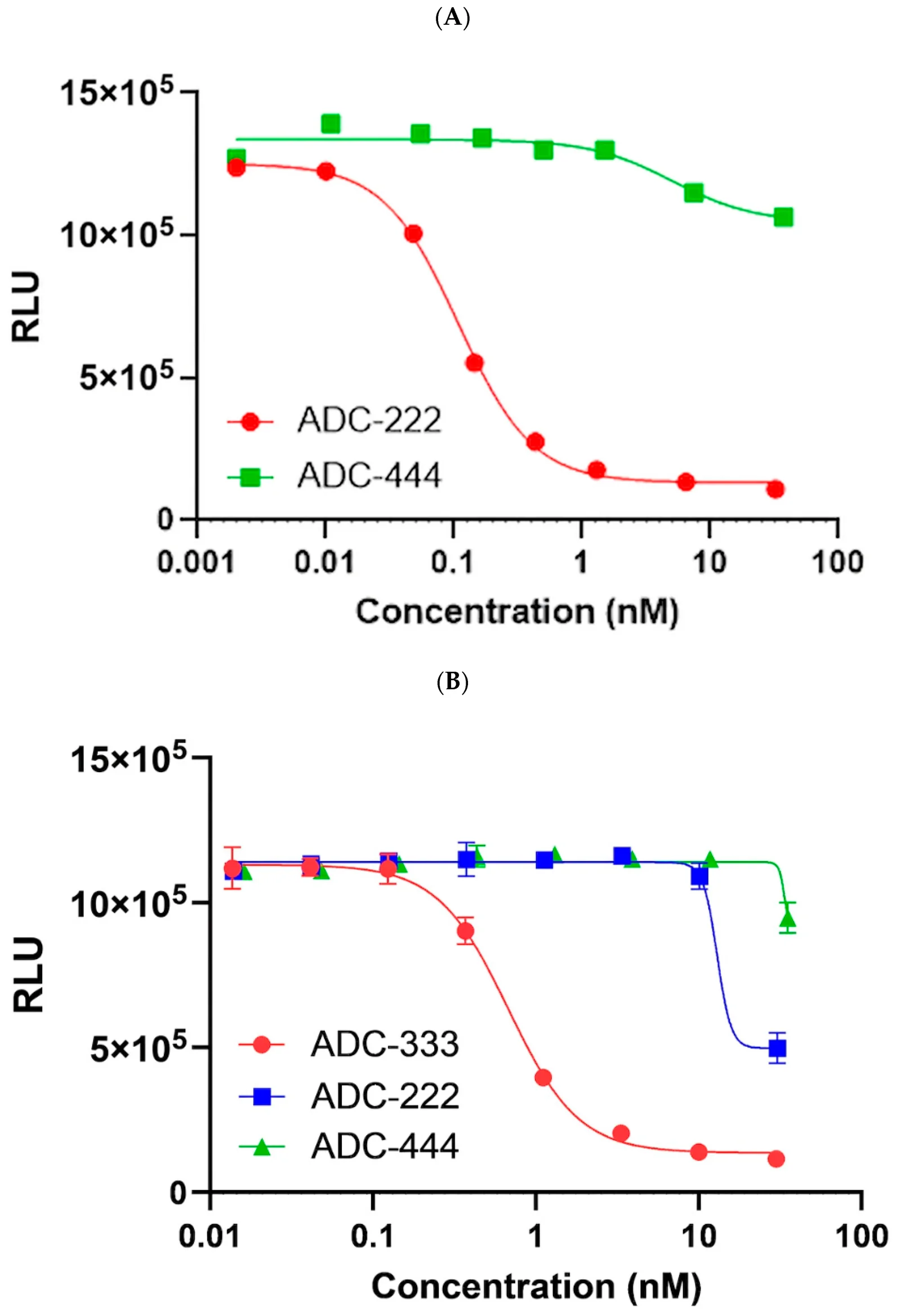
T24 cells engineered with other tumor antigens showed promising CBA. (**A**) Dose–response curves of ADC-222 CBA using T24-Antigen-2+ cells. ADC-444 was included as an isotype ADC. (**B**) Dose–response curve of ADC-333 CBA using T24-Antigen-3+ cells. ADC-222 and ADC-444 were included as isotype ADCs. Error bars represent the standard deviation of N = 2 measurements.

**Table 1:** Comparison of ELISA and CBA Potency.

Stress Condition	Timepoint	CBA		ELISA		DAR	
		%RP	%Control*	%RP	%Control*	Measured	%Control*
AAPH	H0	120	100	125	100	7.8	100
	H6	73	61	91	73	7.5	96
	H24	26	22	23	18	6.8	87
Heat	H1	123	100	122	100	7.7	100
	H24	112	91	128	105	6.7	87
Light	0x	100	100	104	100	7.4	100
	0.5x	15	15	27	26	Cannot integrate	NA

%Control* = Normalized by initial time point.

**Table 2:** Summary of ADC-111 CBA Validation Results.

Performance Characteristics	Acceptance Criteria	Results
Specificity	Non-specific sample must not produce a typical 4-PL curve or fail sample acceptance criteria	Isotype ADC did not produce a typical 4-PL curve
Linearity	Proportional bias falls within ±20% over all potency levels	Proportional bias = −3.5%; For information only: R^2^ = 0.98; Slope = 0.95
Relative Accuracy	90% CI for the %relative bias at each potency level must fall within ±20%	The largest %relative bias was 8.7% at the 35% target potency with 90% CI (4.6, 13.0)
Repeatability	Overall %RSD ≤ 25%	Overall %RSD is 6.9%
Intermediate Precision	Overall %RSD ≤ 25%	Overall %RSD is 7.5%
Range	The range is established by successfully meeting the acceptance criteria for Accuracy, Linearity, and Precision over all runs	The assay range is confirmed as 35–200%

**Table 3:** Comparison of ADC-111 CBA using CC and RTU.

	CC			RTU		
Target %RP	%RP	%GSD	%Recovery	%RP	%GSD	%Recovery
50	47	2	94	49	2	98
150	147	5	98	159	3	106
200	210	5	105	202	3	101

## Data Availability

The original contributions presented in this study are included in the article. Further inquiries can be directed to the corresponding author.
